# Author Correction: GFI1 tethers the NuRD complex to open and transcriptionally active chromatin in myeloid progenitors

**DOI:** 10.1038/s42003-022-03822-x

**Published:** 2022-09-07

**Authors:** Anne Helness, Jennifer Fraszczak, Charles Joly-Beauparlant, Halil Bagci, Christian Trahan, Kaifee Arman, Peiman Shooshtarizadeh, Riyan Chen, Marina Ayoub, Jean-François Côté, Marlene Oeffinger, Arnaud Droit, Tarik Möröy

**Affiliations:** 1 Institut de recherches cliniques de Montréal, Montréal, QC H2W 1R7 Canada; 2 Département de Médecine Moléculaire, Faculté de Médecine, Université Laval, Québec, QC Canada; 3 Department of Anatomy and Cell Biology, McGill University, Montréal, QC H3A 0C7 Canada; 4 Département de Biochimie, Université de Montréal, Montréal, QC H3C 3J7 Canada; 5 Division of Experimental Medicine, McGill University, Montreal, QC Canada; 6 Département de Microbiologie, Infectiologie et Immunologie, Université de Montréal, Montréal, QC Canada; 7 Present Address: Institute for Biochemistry, ETH Zürich, Zürich, Switzerland; 8 Present Address: Hôpital pour Enfants, Ste Justine, Montreal, QC Canada

**Keywords:** Molecular biology, Chromatin

Correction to: *Communications Biology* 10.1038/s42003-021-02889-2, published online 02 December 2021.

The original version of this Article mistakenly referred to “anti-H3K9me2 (ab1220; AbCam)” when describing the Chromatin immunoprecipitation (ChIP) protocol in the Methods. The correct version of this text has now been corrected to reference “anti-H3K9me3 (ab8898; AbCam)”.

The original version of this Article was also missing the accession information for H3K4me1 ChIP-seq datasets in the Data Availability statement:

“Previously published ChIP-seq and ATAC-Seq data, which are presented in Fig. 4 and Suppl. Figure 4, are available under the following accession numbers: H3K27ac (GSM1441273), H3K27me3 (DRR023959), H3K9me3 (DRR023962), and ATAC-Seq (DRR023962).”

The correct statement now reads:

“Previously published ChIP-seq and ATAC-Seq data, which are presented in Fig. 4 and Suppl. Figure 4, are available under the following accession numbers: H3K27ac (GSM1441273), H3K27me3 (DRR023959), H3K9me3 (DRR023962), H3K4me1 (GSM1441289), and ATAC-Seq (DRR023962).”

Both errors have now been corrected in the PDF and HTML versions of the Article.

