# Supplementary material for: GFI1 tethers the NuRD complex to open and transcriptionally active chromatin in myeloid progenitors
Source: Commun Biol. 2021 Dec 2;4:1356. doi: 10.1038/s42003-021-02889-2 (PMC8639993; doi:10.1038/s42003-021-02889-2)
Supplement: Supplementary file 2 — Supplementary Information [file 42003_2021_2889_MOESM2_ESM.pdf]

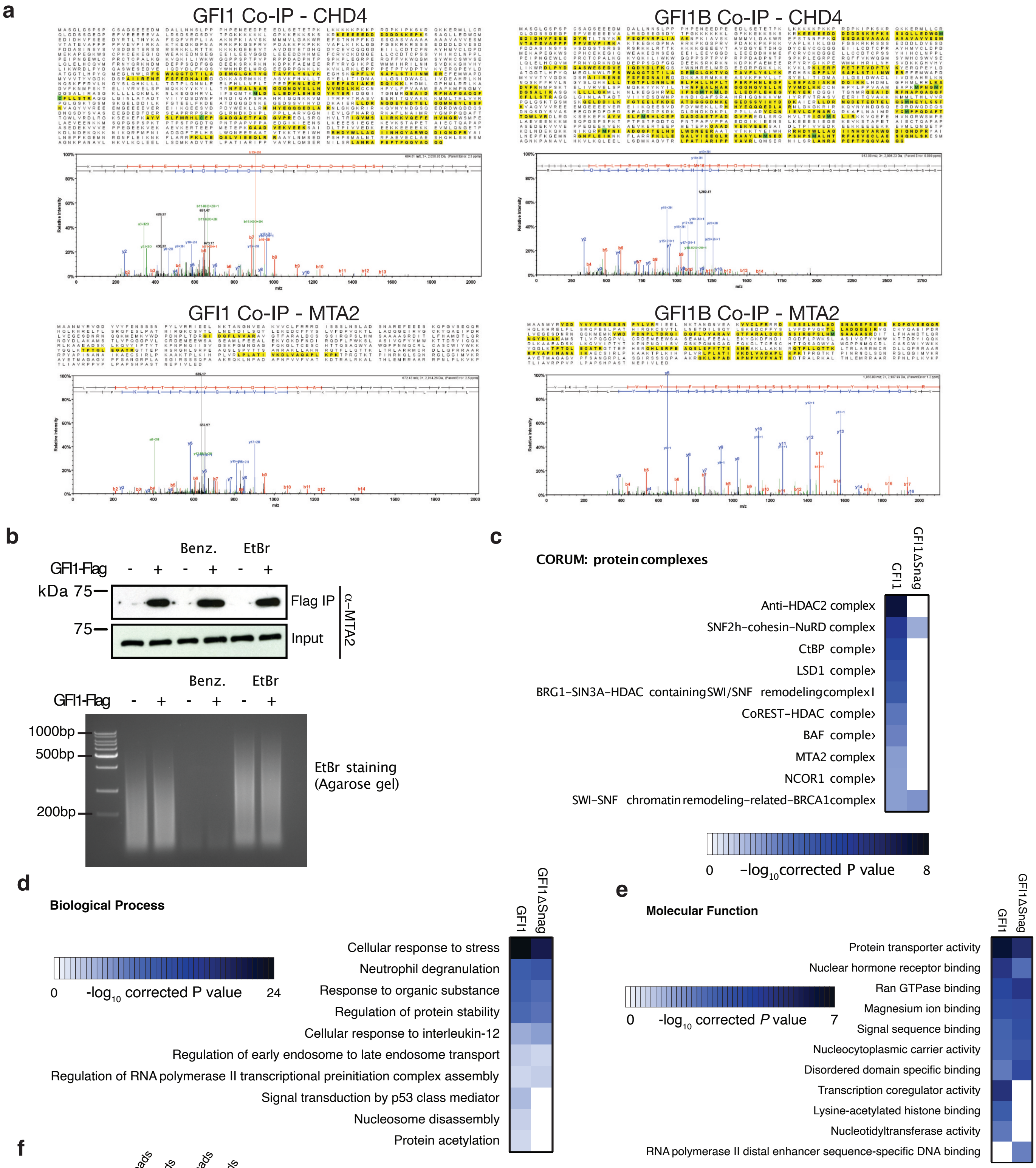

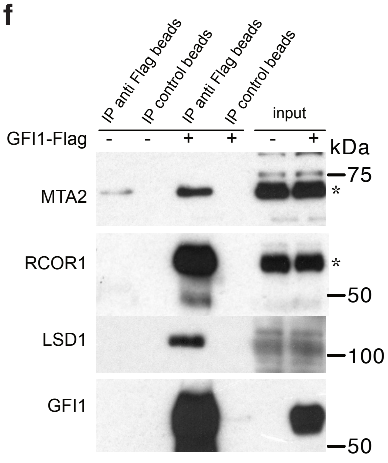

Suppl. Figure 1:

a) Amino acid sequences of CHD4 and MTA2 with peptides identified through mass spectrometry highlighted in yellow with the corresponding spectra below.

b) Immunoprecipitation experiments with cells expressing Flag tagged GFI1 to detect MTA2 in the presence of absence of Benzonase

c) - e) Heatmaps showing CORUM terms, Biological Processes and Molecular Functions of associated protein complexes, respectively, of related GO terms in Flp-In T-REx HEK293T cells expressing GFI1WT-BirA*-Flag or GFI1ΔSNAG-BirA*-Flag. GO term or CORUM protein complex enrichment scores are shown as the -log10 of corrected P values, illustrated by different color intensities.

f) GFI1-Flag fusion protein was immunoprecipitated from HEK293T cells with M2 Anti-agarose beads or noncoupled beads alone; precipitates were separated by SDS–PAGE and blotted for the indicated proteins.

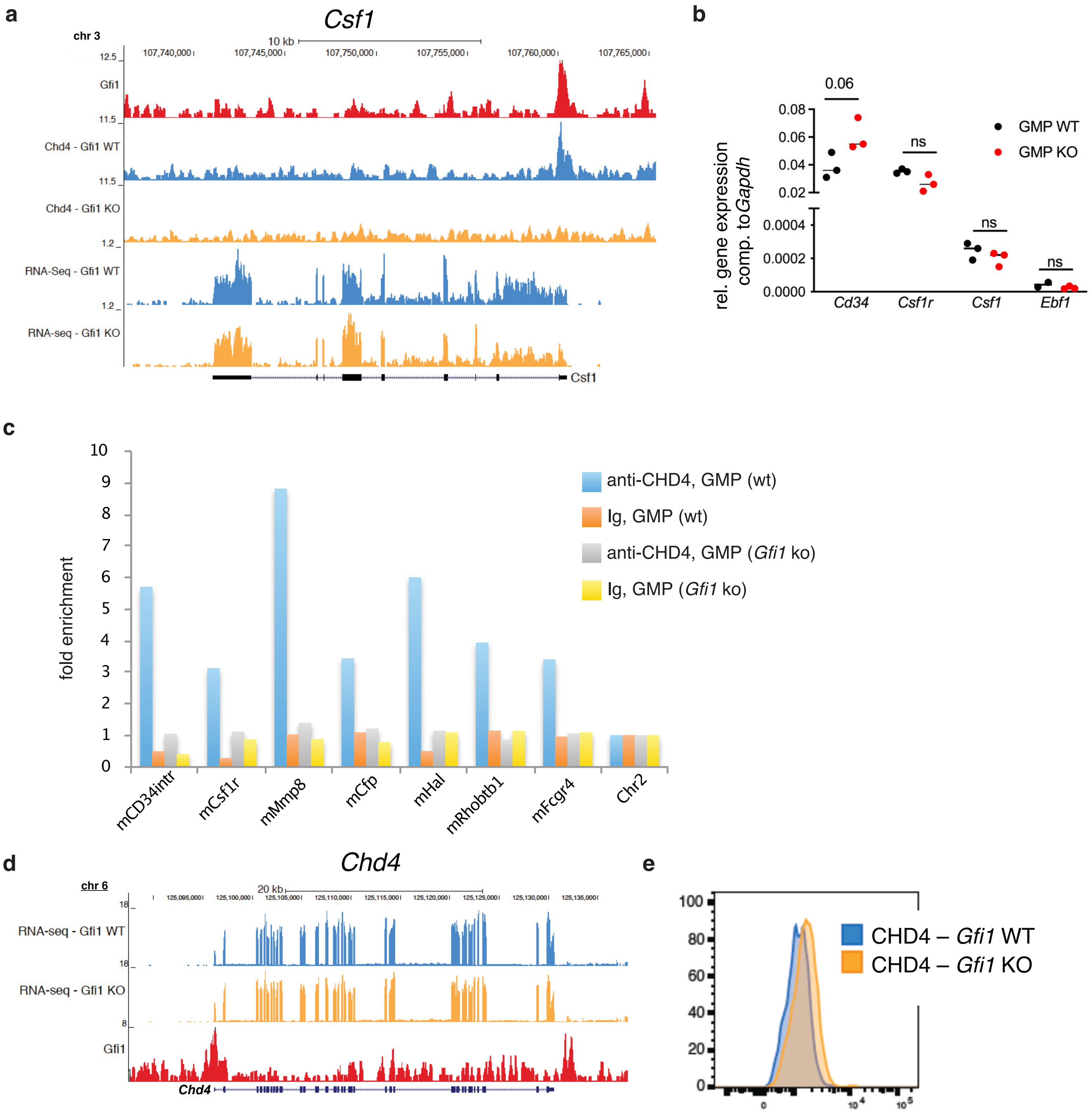

Suppl. Figure 2:
a) Schematic depiction of the *Csf1* locus encoding M-CSF. Shown is the enrichment of reads after ChIP-seq with antibodies against GFI1 or CHD4 in GMPs from either WT or *Gfi1*KO mice and the enrichment of reads after an RNA-seq experiment from WT of *Gfi1* KO GMPs. The transcription start site is indicated (TSS). Annotations represent locations on the mouse genome version GRCm38 (mm10).
b) Expression measured by RT-QPCR of the indicated genes in GMPs from *Gfi1* wt and *Gfi1* knockout (KO) animals; (n = 3 for each genotype).
c) ChIP-qPCR for CHD4 in one sample of primary *Gfi1* WT and one sample of primary *Gfi1* KO GMPs of seven exemplary genes selected from the group of 841 loci where CHD4 binding was lost in the absence of GFI1.
d) RNA-seq profile of the *Chd4* gene in GMPs from WT or *Gfi1* KO mice. Annotations represent locations on the mouse genome version GRCm38 (mm10).
e) Flow cytometric profile for CHD4 (intracellular staining) in GMPs from WT or *Gfi1* KO mice.

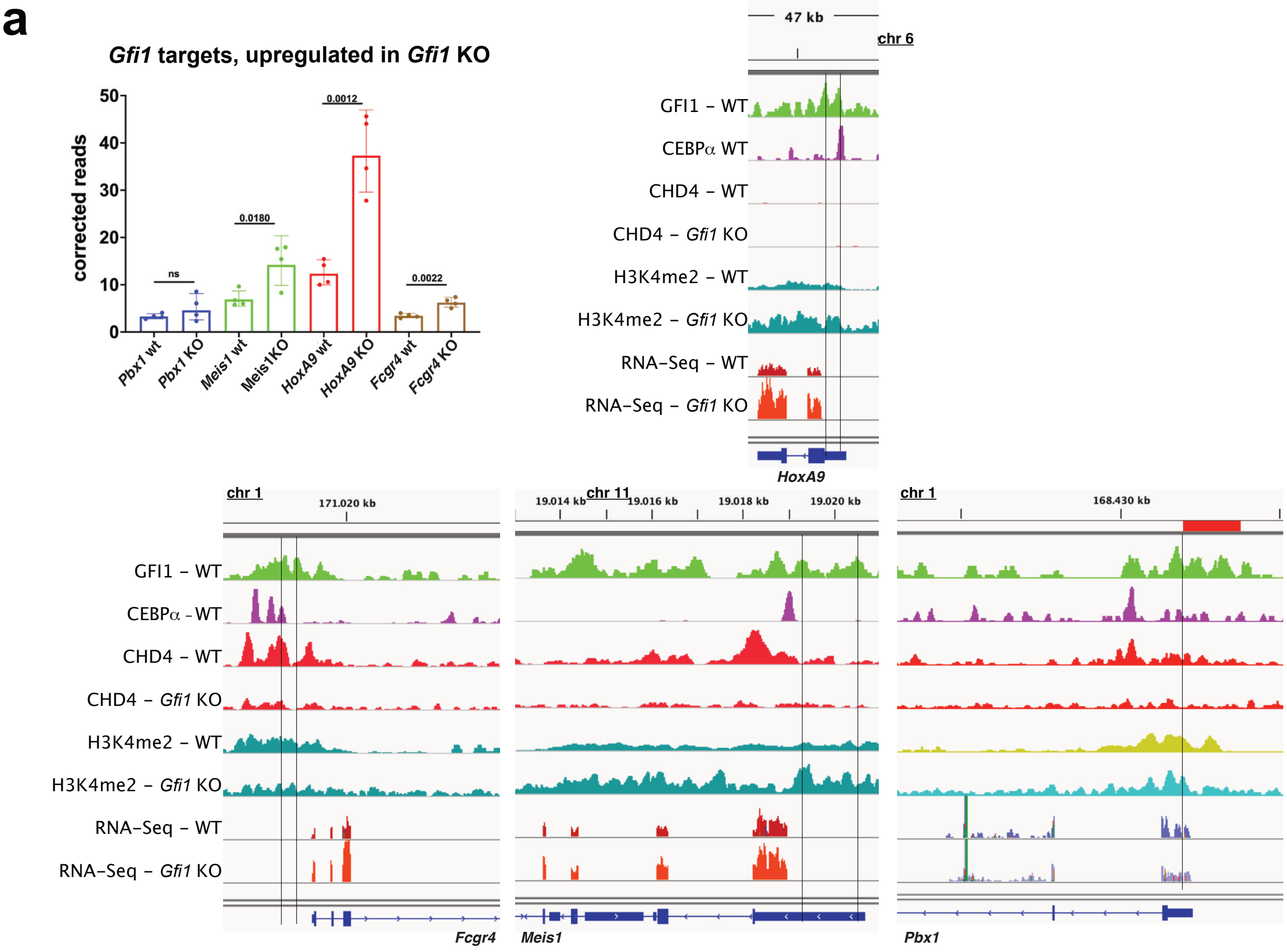

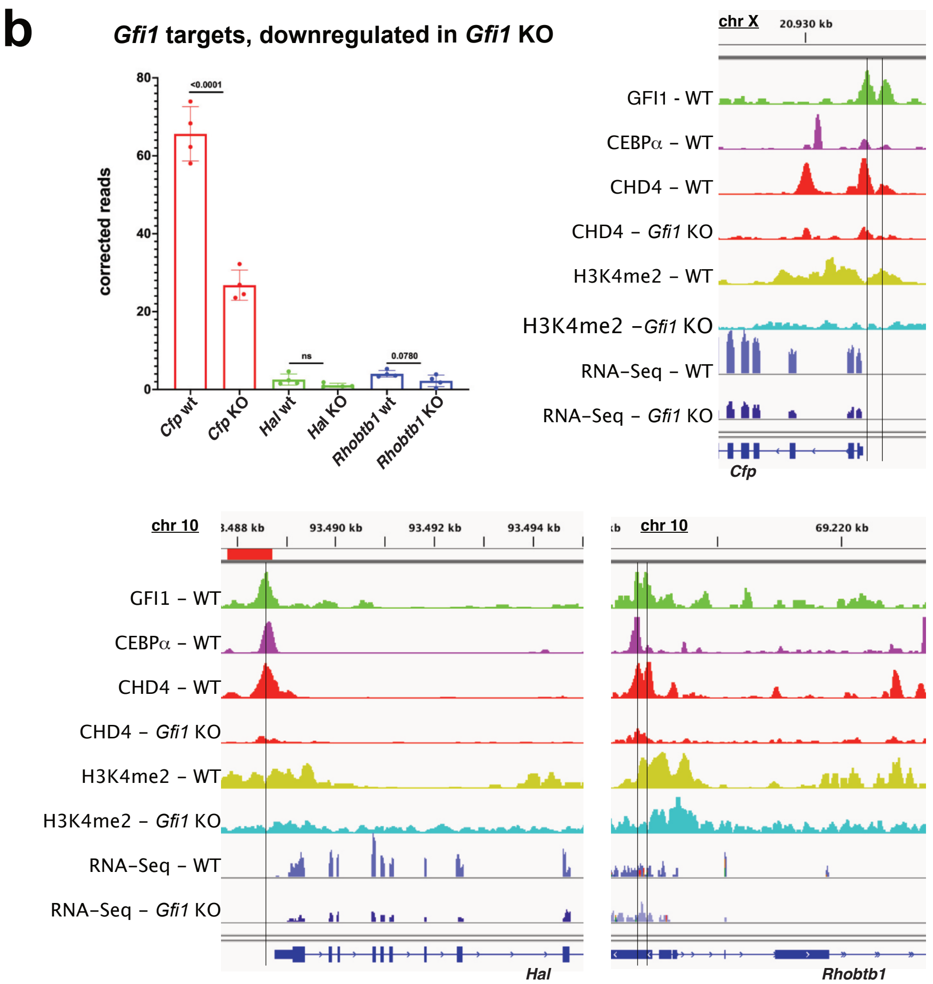

Suppl. Figure 3
Compilation of RNA-seq and ChIP seq data for known GFI1 target genes that are either upregulated **(a)** or downregulated **(b)** in *Gfi1* KO GMPs. RT-PCR data were compiled from four indepdent RNA-seq analyses performed with GMPs from wt or *Gfi1* KO animals as indicated; p-values were calculated using a t-test and are indicated. Annotations represent locations on the mouse genome version GRCm38 (mm10).

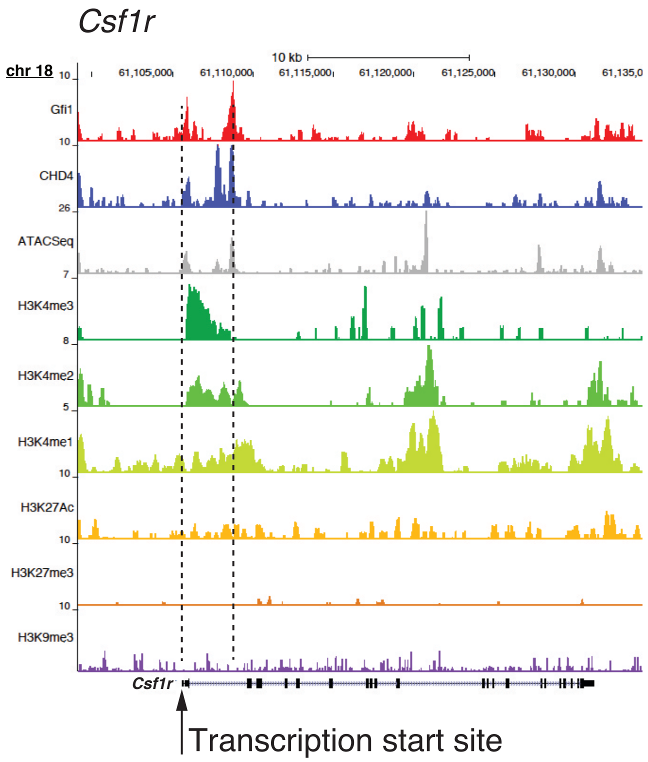

Suppl. Figure 4
Depiction of the ATAC-Seq and ChIP-Seq data at the GFI1 target gene *Csf1*. Indicated are the tracks corresponding to the individual experiments, the gene and the TSS. Annotations represent locations on the mouse genome version GRCm38 (mm10).

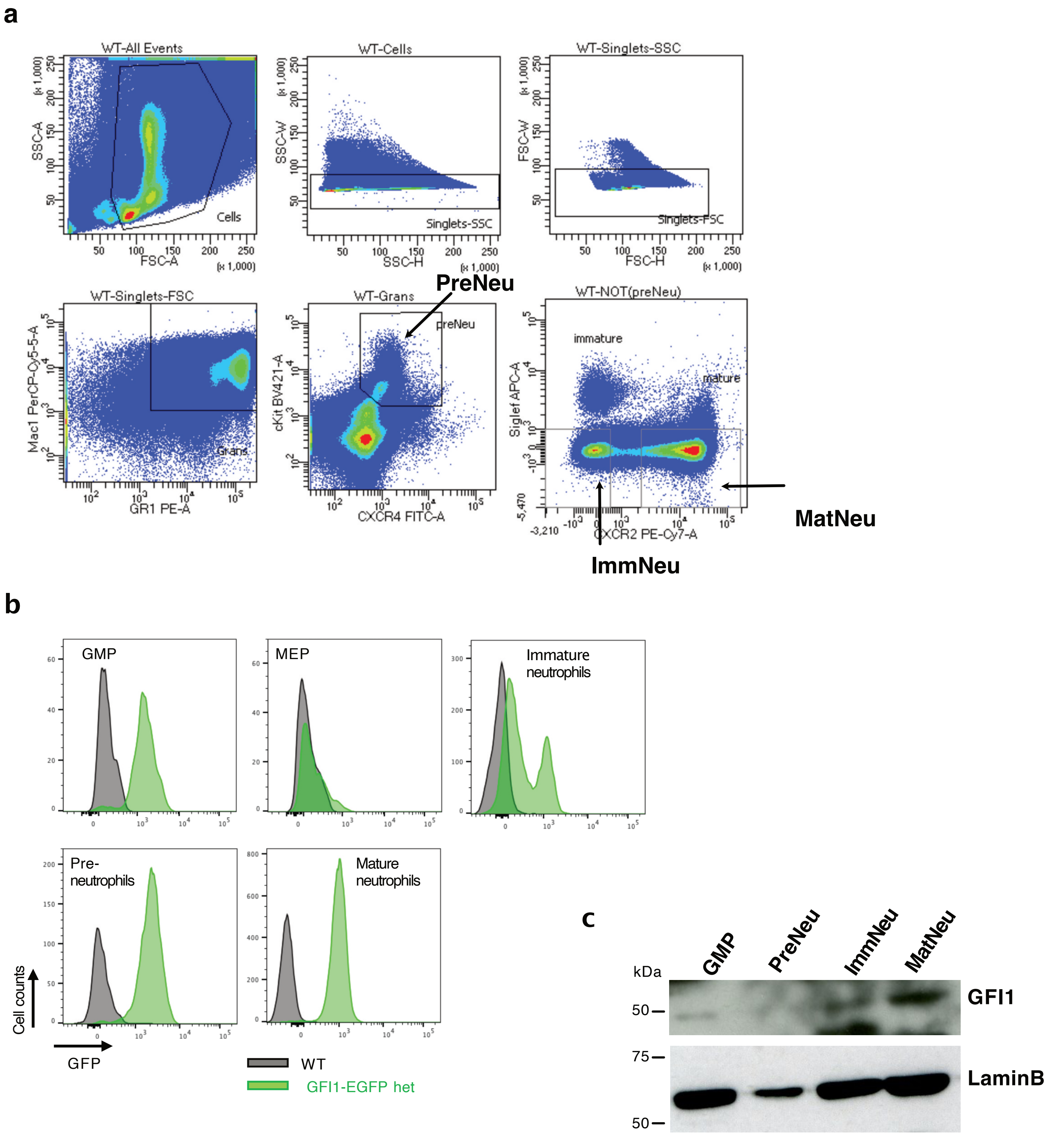

Suppl. Figure 5

Gfi1:GFP expression in cellular subsets of the neutrophil lineage

a) Flow cytometric sorting strategy for pre-neutrophils (preNeu), immature Neutrophils (immatNeu) and mature Neutrophils (matNeus) cells according to the following markers: preNeu: MAC-1$^+$GR1$^+$cKIT$^+$CXCR4$^+$, immNeu: Mac1$^+$Gr-1$^+$CXCR4$^-$cKIT$^-$SiglecF$^-$CXCR2$^-$, matNeu: MAC-1$^+$GR1$^+$CXCR4$^-$cKit$^-$SiglecF$^-$CXCR2$^+$, see Ref 44.

b) GFP intensity of the neutrophil lineage cell sub populations of total bone marrow from heterozygous *Gfi1*:GFP knockin mice, expressing a GFP cDNA under the control of the *Gfi1* promoter (ref 45). GMP: lin$^-$ cKIT$^+$SCA1$^-$CD16/32$^+$CD34$^+$, Pre-neutrophils: Gr-1$^+$MAC-1$^+$Ckit$^+$CXCR4$^+$, immature Neutrophils: GR1$^+$MAC-1$^+$cKIT$^-$ CXCR4$^-$SIGLECF$^-$CXCR2$^-$, Mature Neutrophils: GR1$^+$MAC-1$^+$cKIT$^-$CXCR4$^-$SIGLECF$^-$CXCR2$^+$, MEP (lin$^-$cKIT$^+$SCA1$^-$ CD16/32$^-$CD34$^+$), which do not express GFI1 (Ref 40), are used as a control.

c) Western blot for GFI1 and Lamin expression using nuclear extracts from the cell populations indicated in **a)**

a

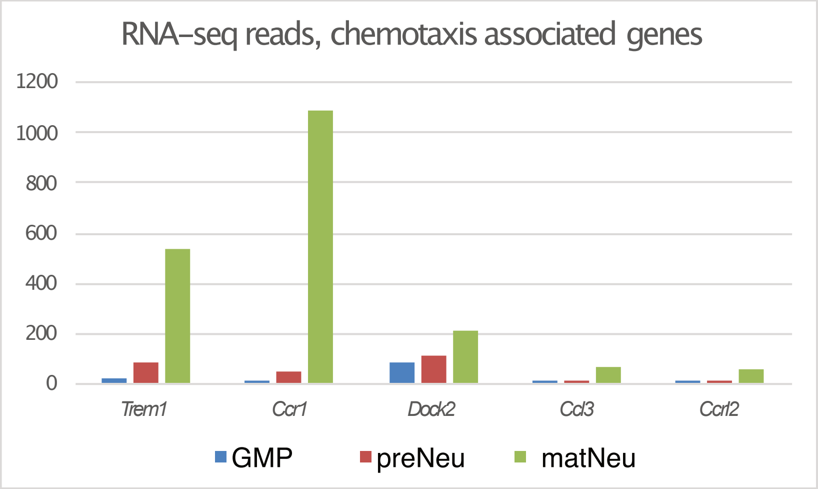

b

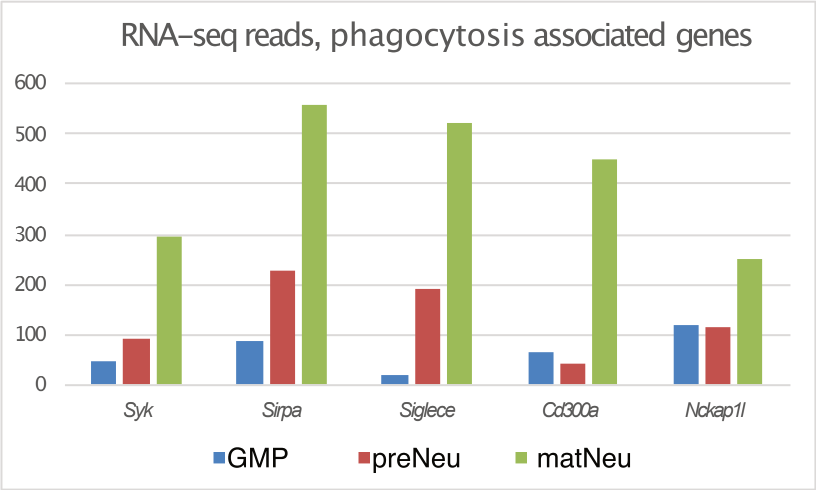

Suppl. Fig. 6
a), b) RNA-seq normalized reads of genes associated with chemotaxis or phagocytosis according to ref. 44.

a

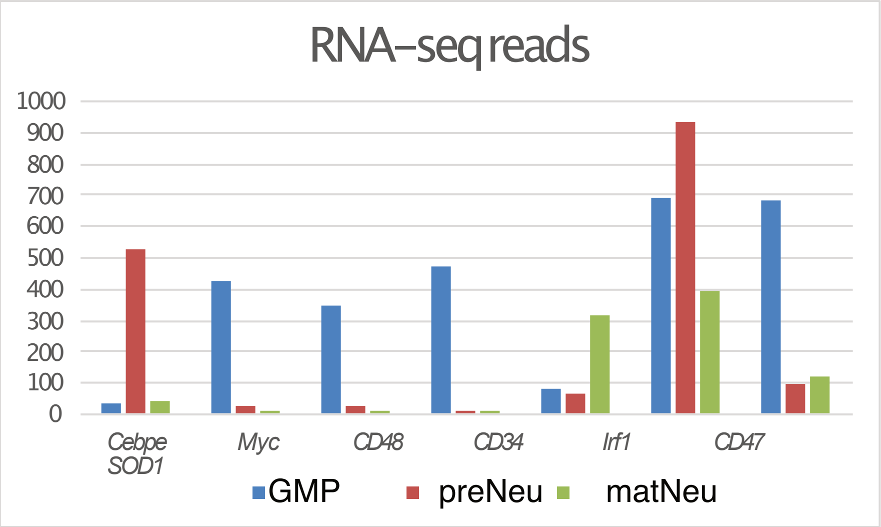

b

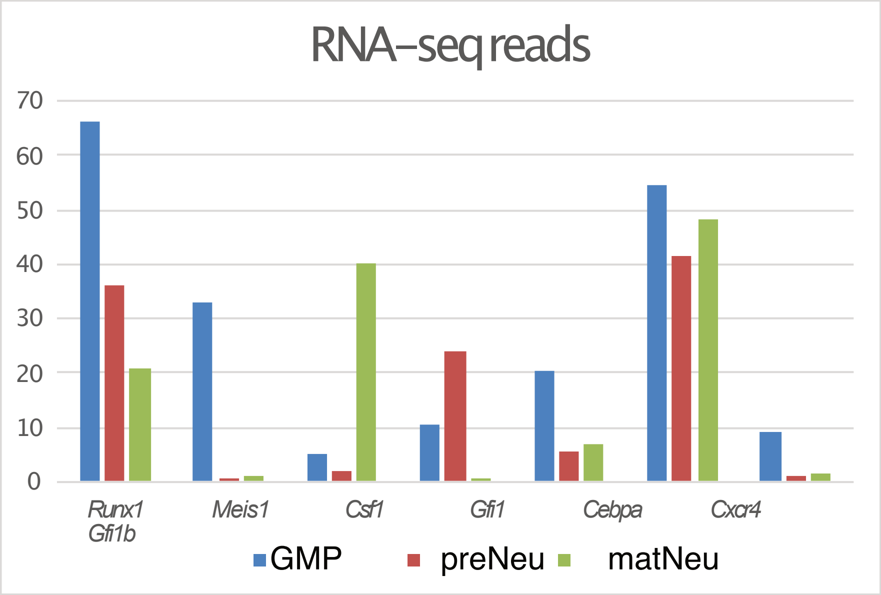

C

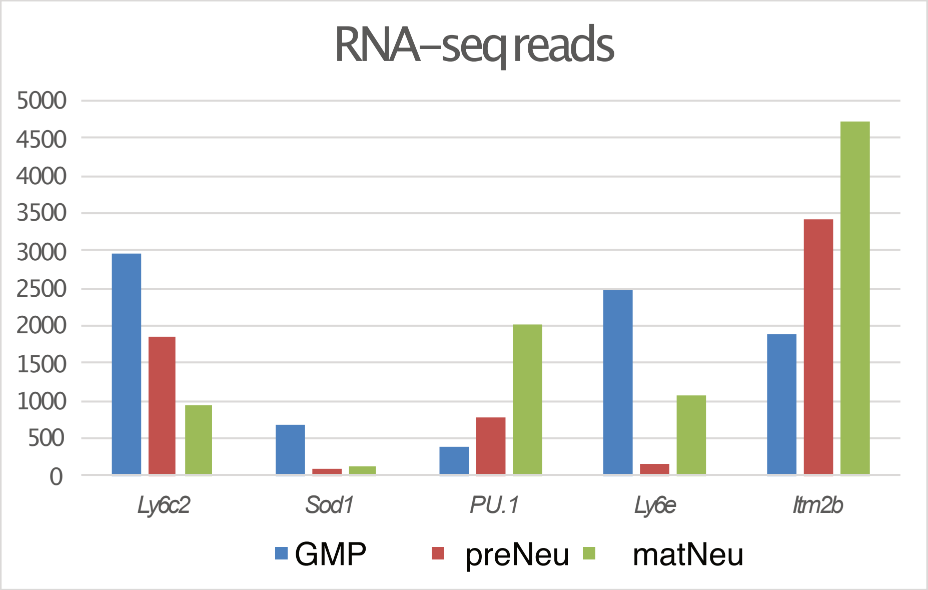

Suppl. Fig. 7
a) - c) RNA-seq normalized reads of genes associated with myeloid differentiation according to Ref 44.

Suppl. Fig. 8
Whole Western blots for Fig. 2a, b

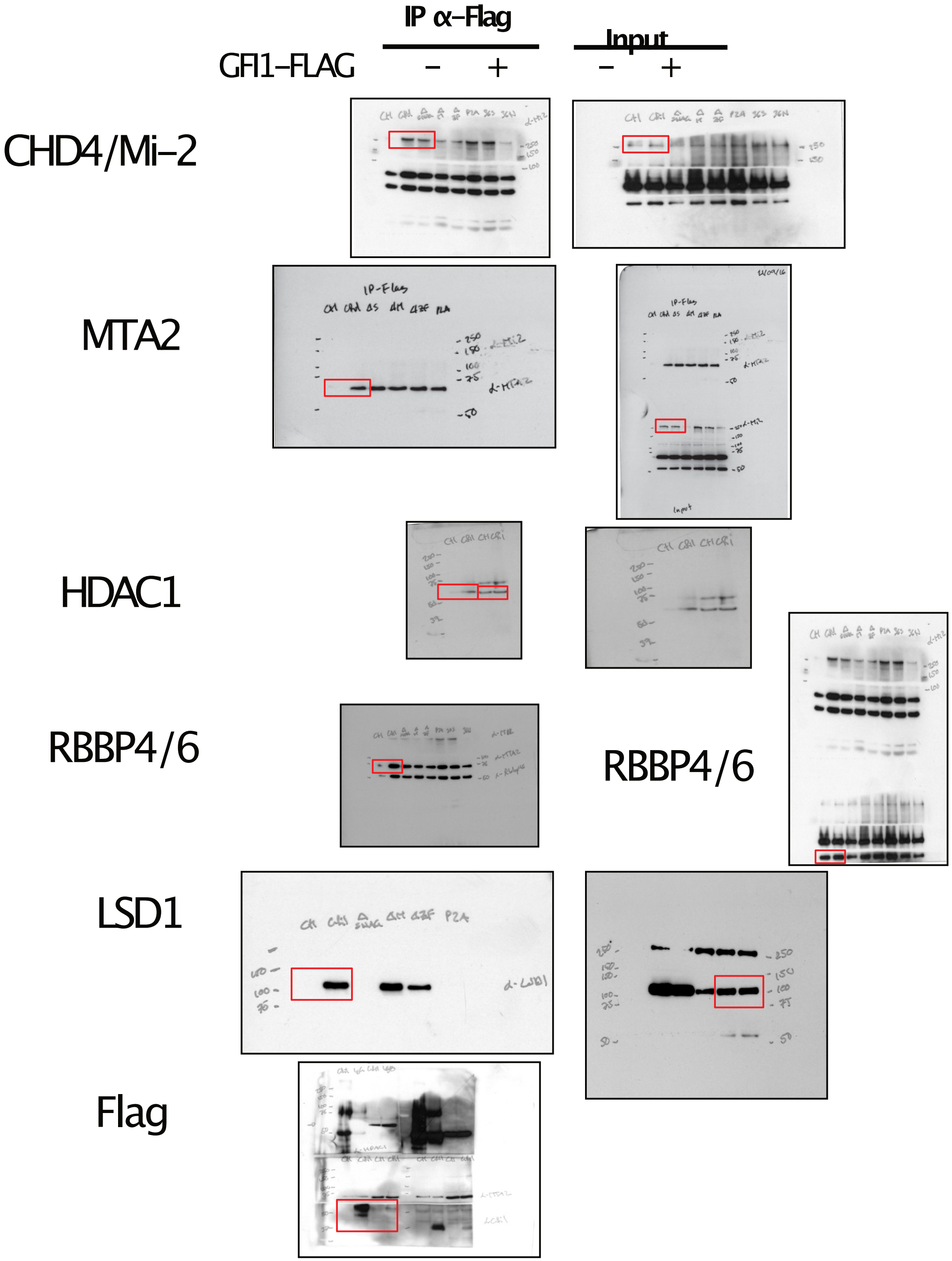

Suppl. Fig. 8
Whole Western blots for Fig. 2a, b

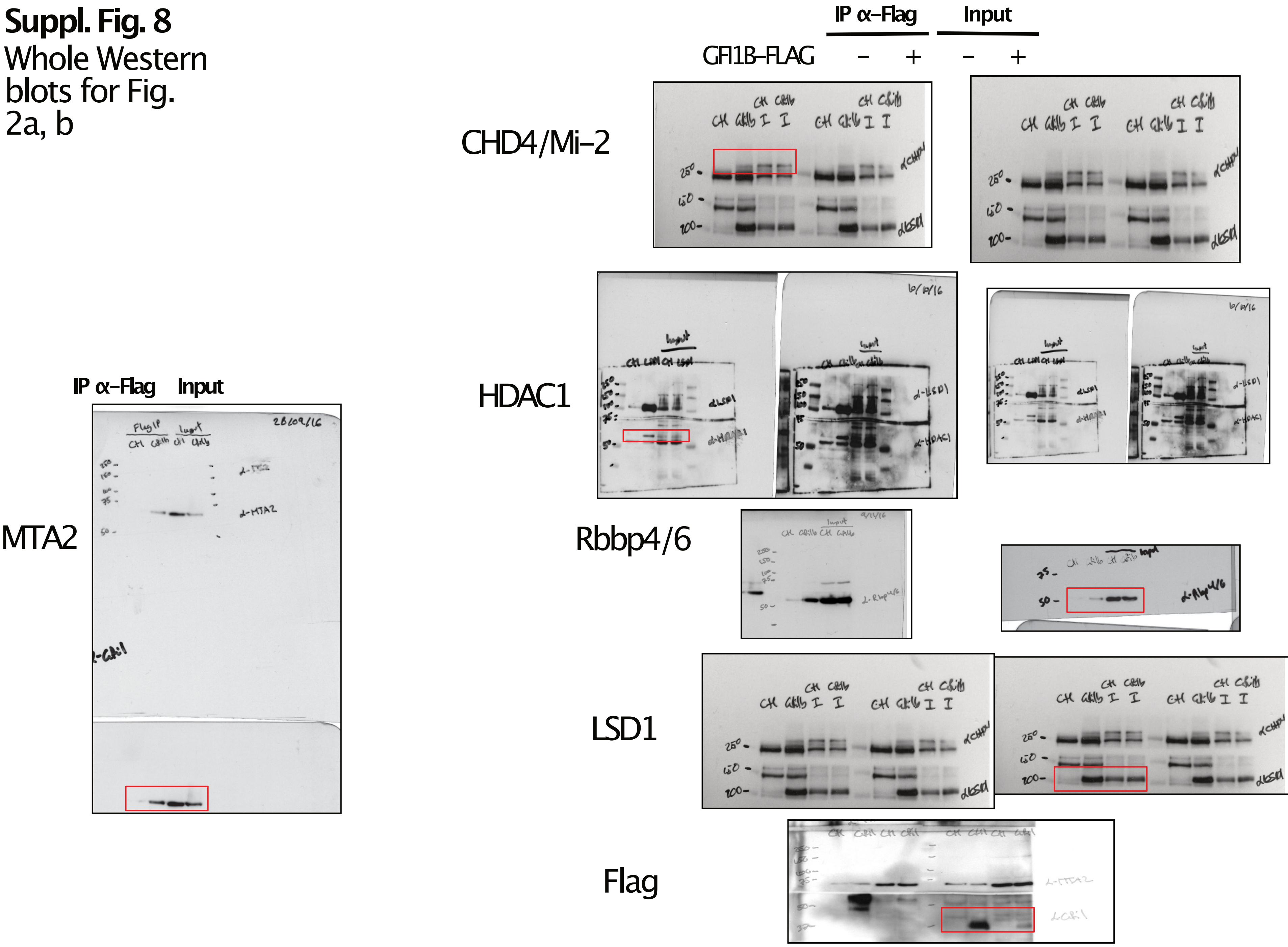

Suppl. Fig. 8
Whole Western blots for Fig. 2a, b

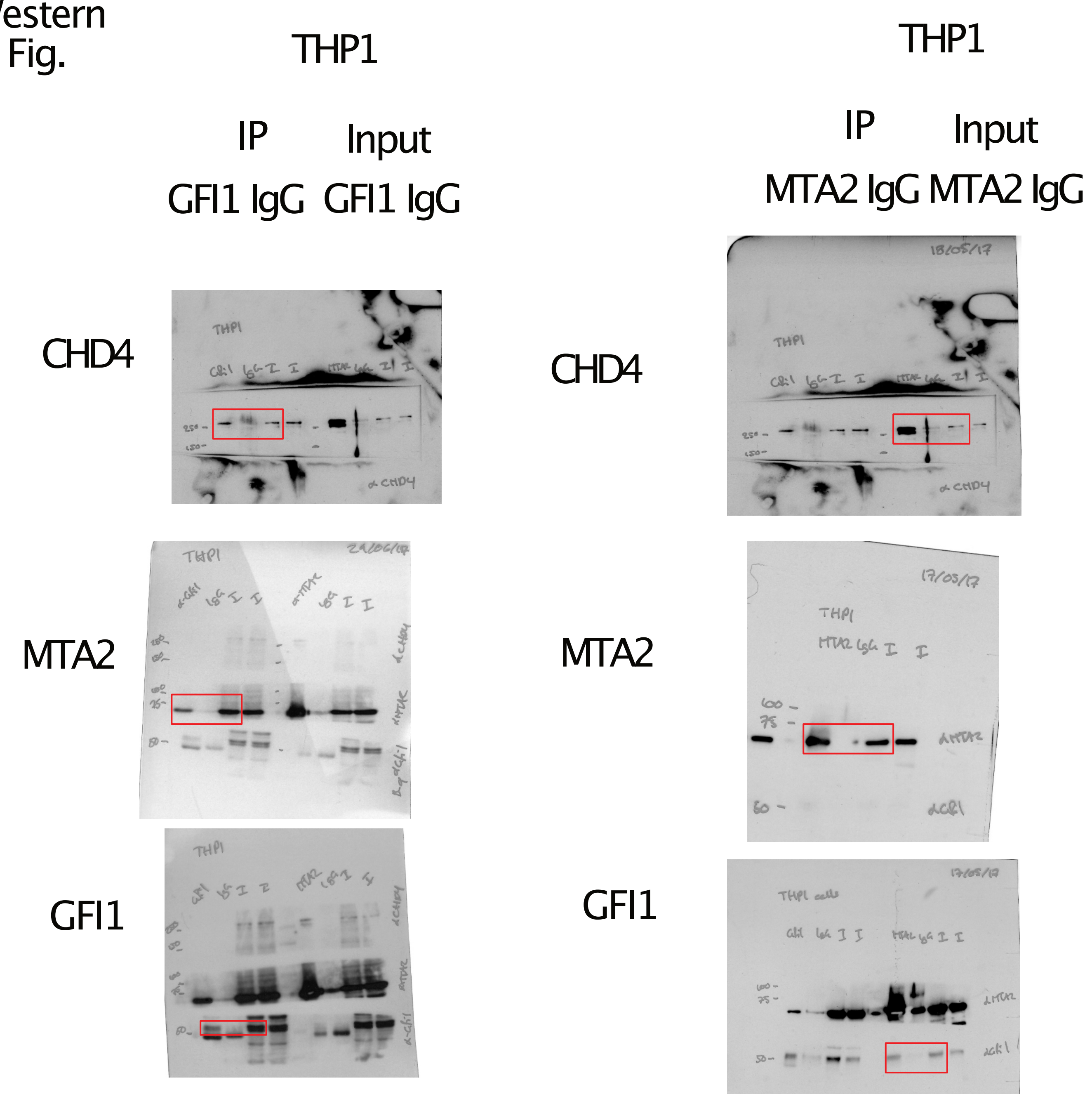

Suppl. Fig. 8
Whole Western blots for Fig. 2c

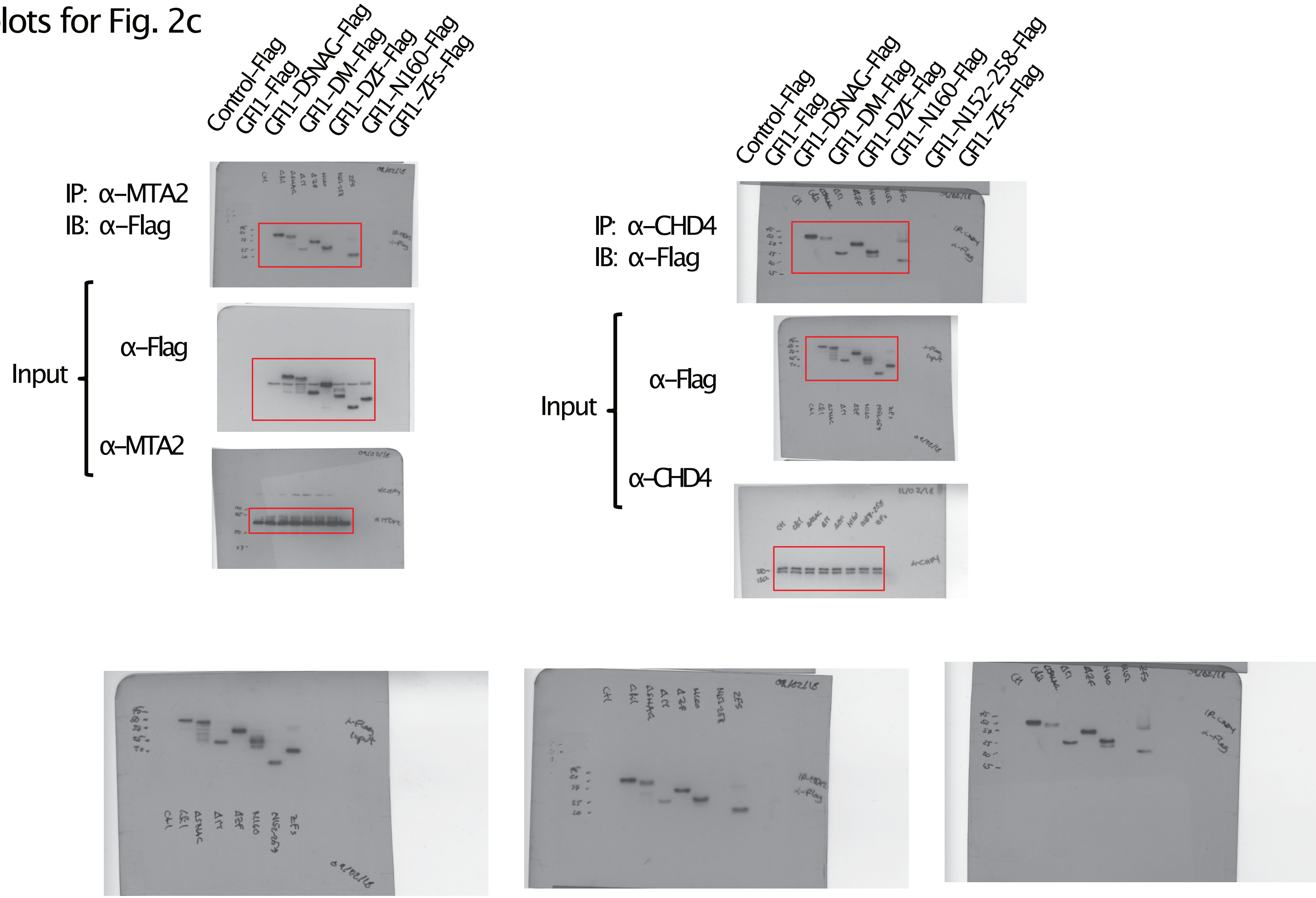

Suppl. Fig. 8
Whole Western blots for Fig. 2e

GFI1

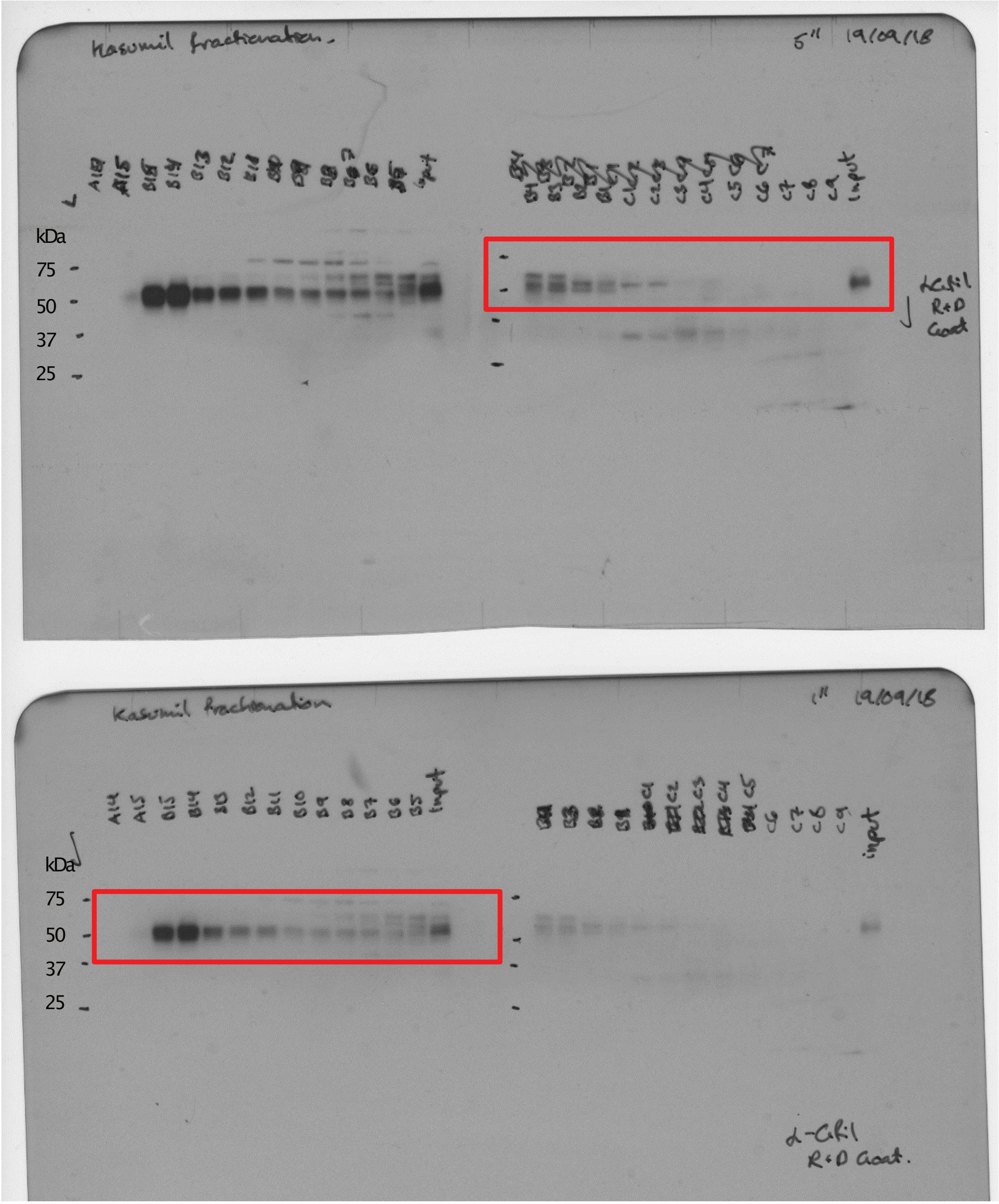

Suppl. Fig. 8
Whole Western blots for Fig. 2e

CHD4

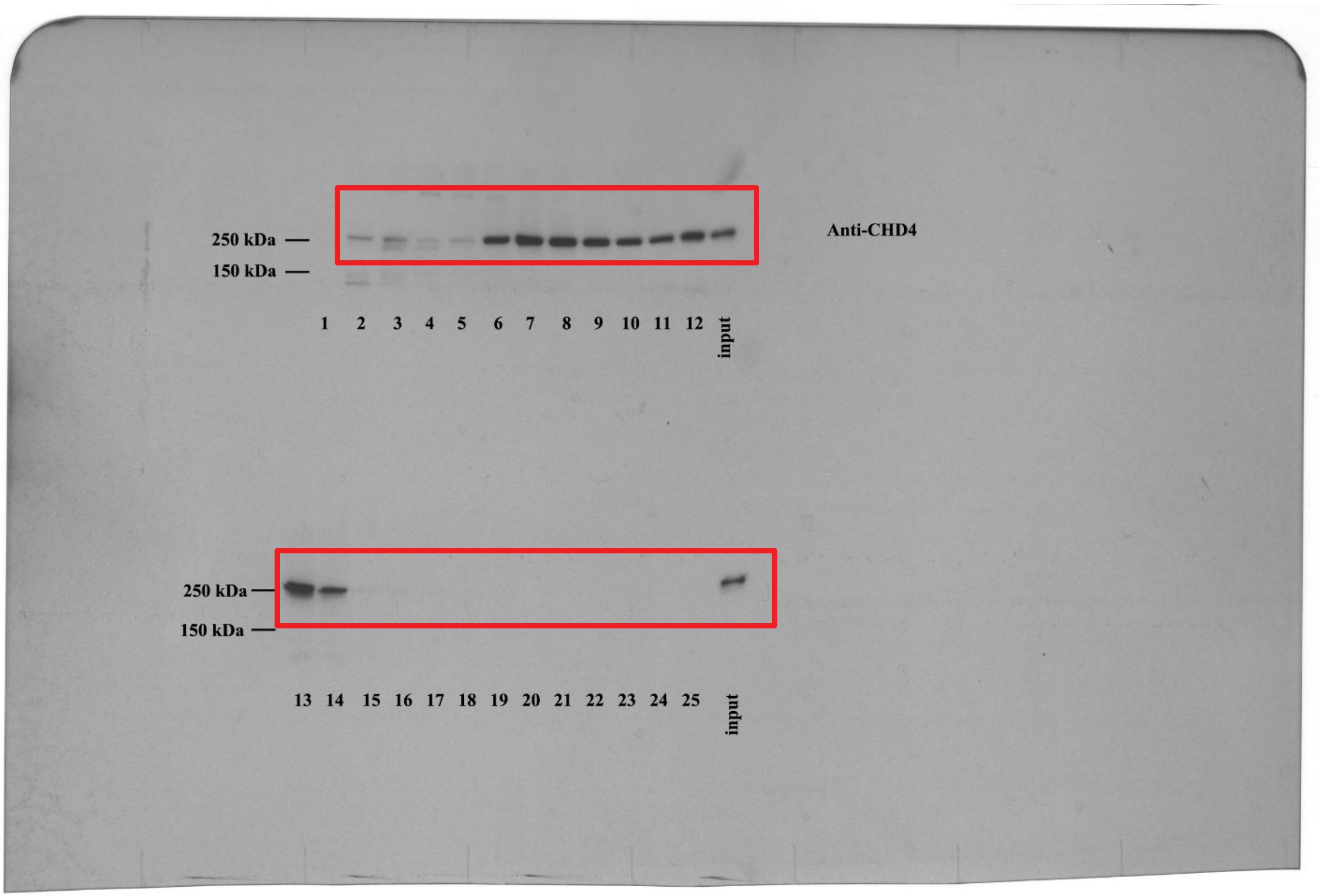

Suppl. Fig. 8

Whole Western blots for Fig. 2e

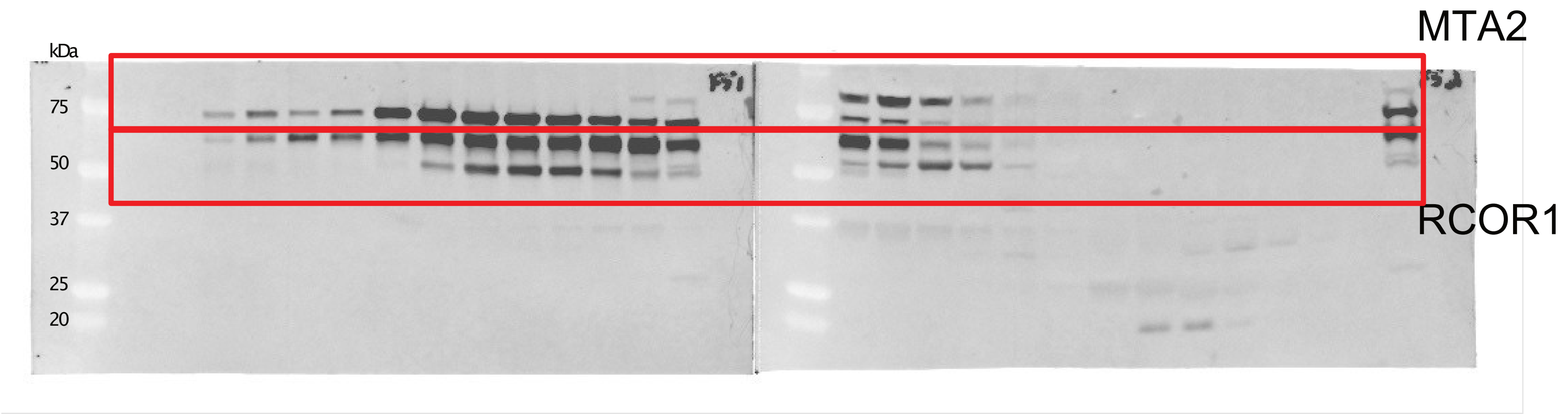

Suppl. Fig. 8

Whole Western blots for Fig. 2e

LSD1

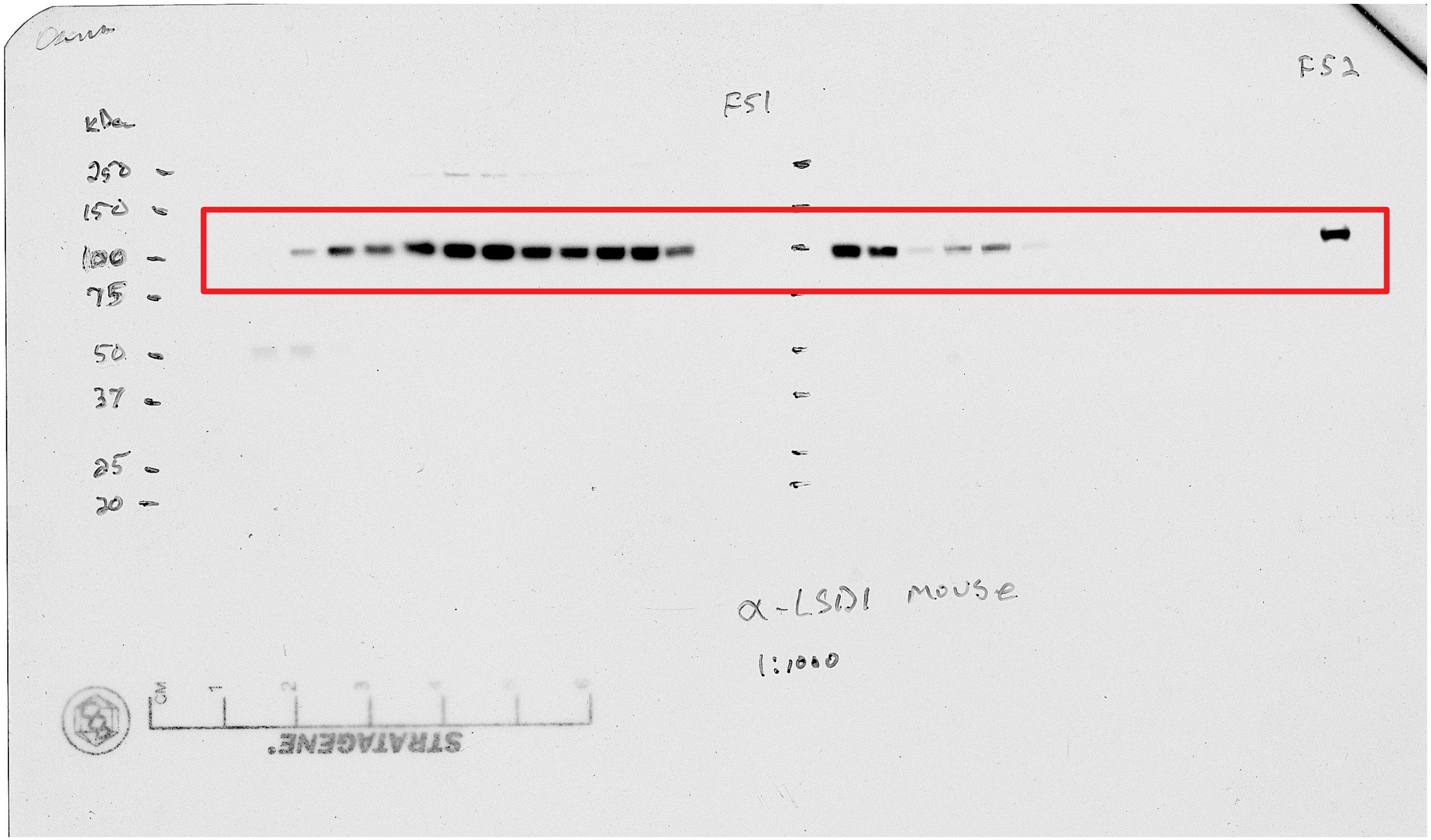

Suppl. Fig. 8

Whole Western blots for Fig. 2f

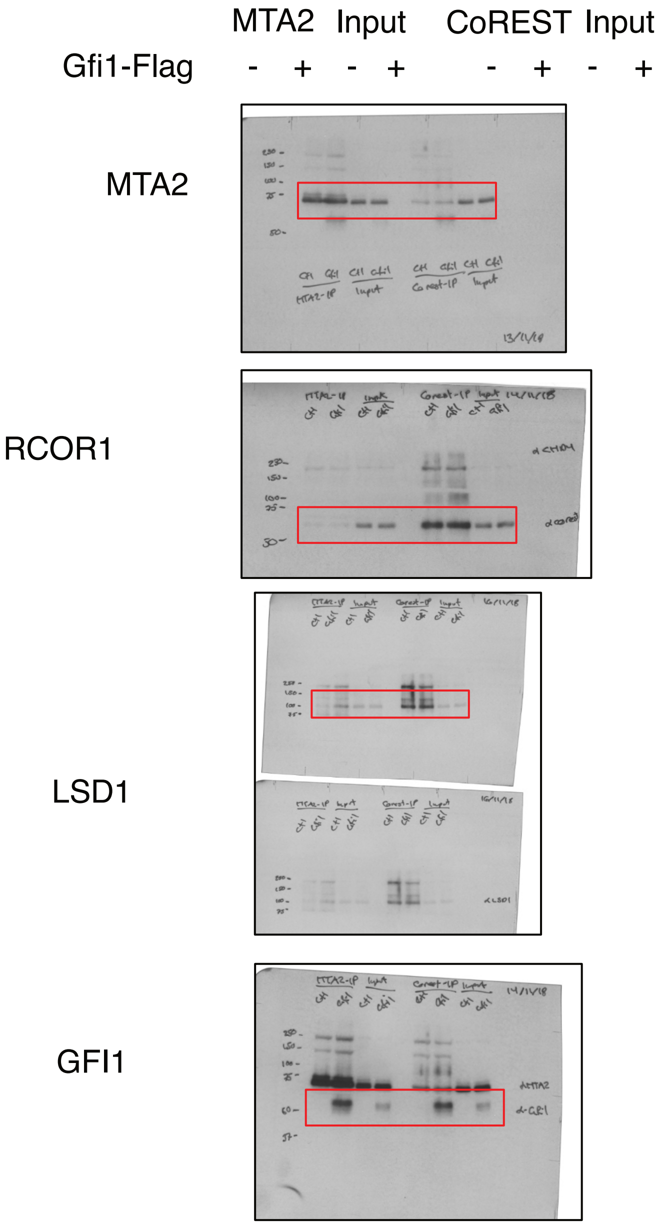

Suppl. Fig. 9
Whole Western blots for suppl. Fig. 1c

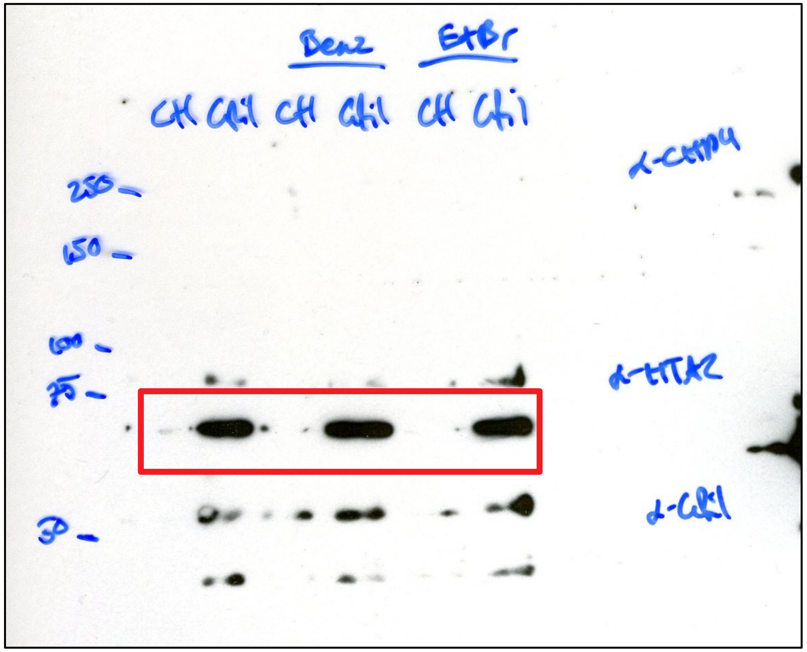

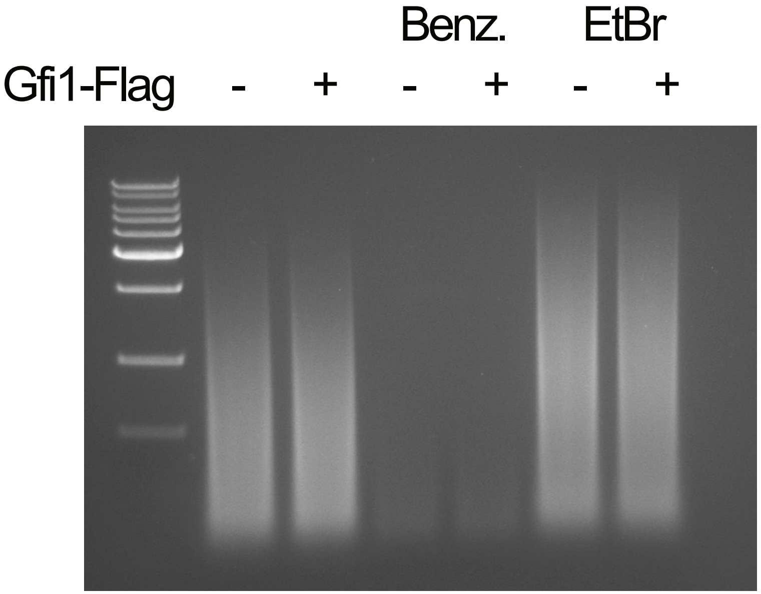

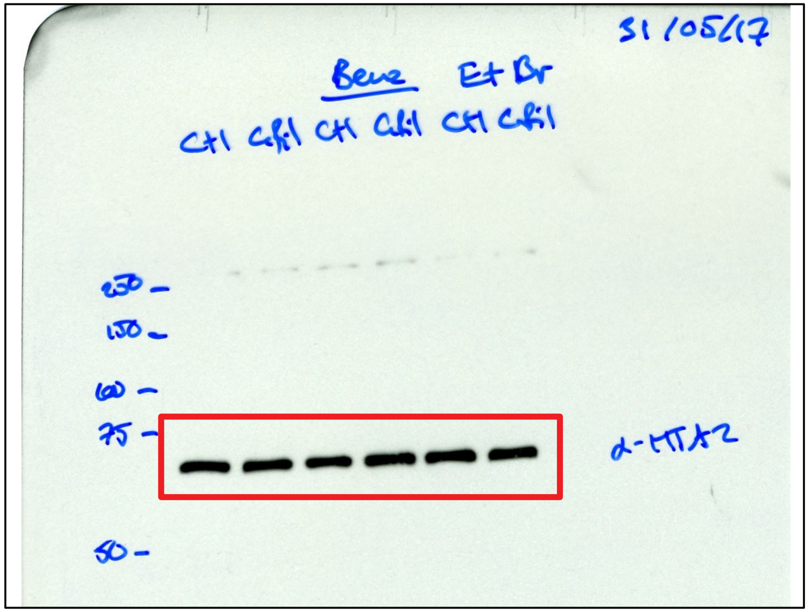

Suppl. Fig. 10
Whole Western blots for suppl. Fig. 1f

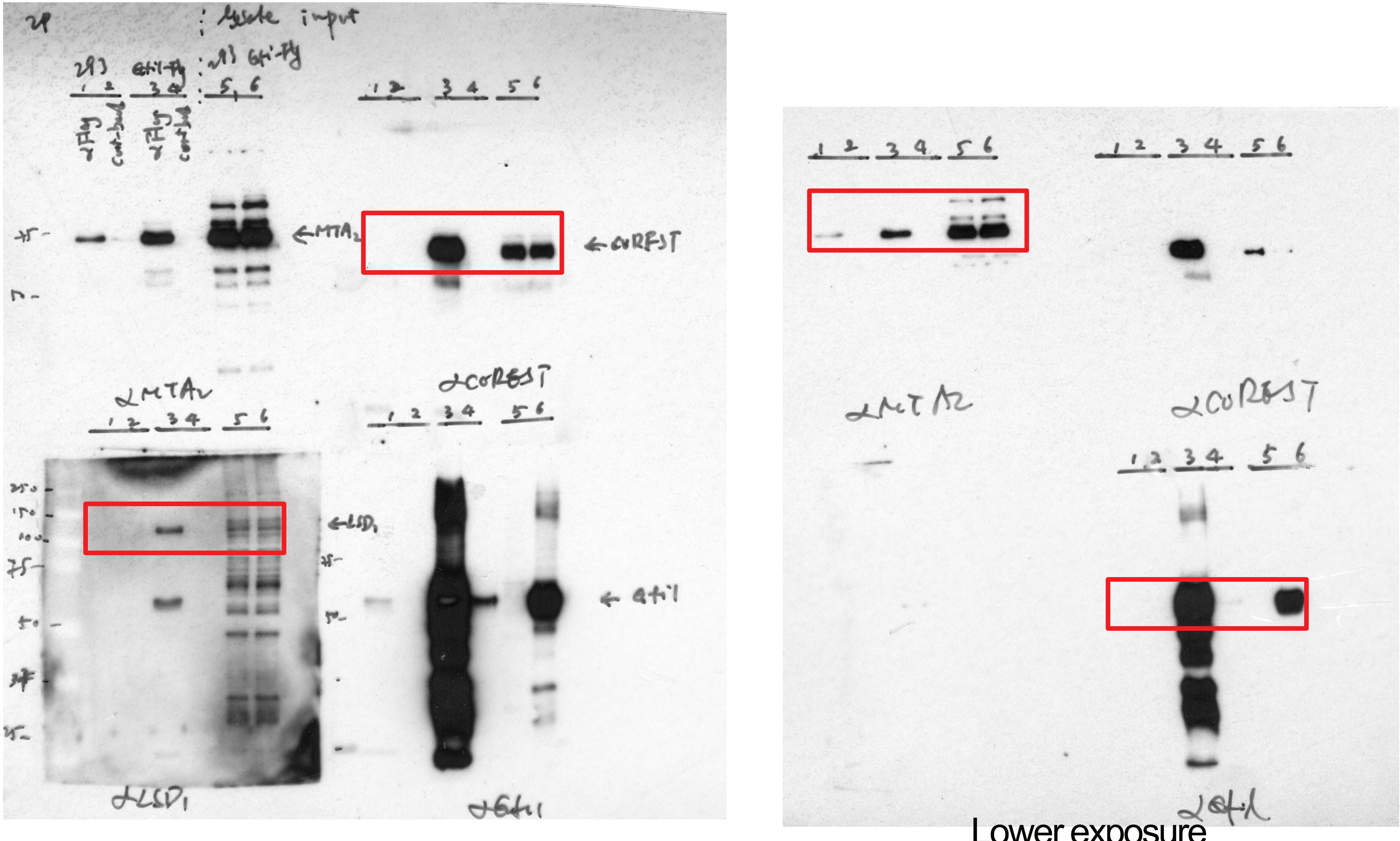

Suppl. Fig. 11

Gating strategy for isolating GMPs from bone marrow

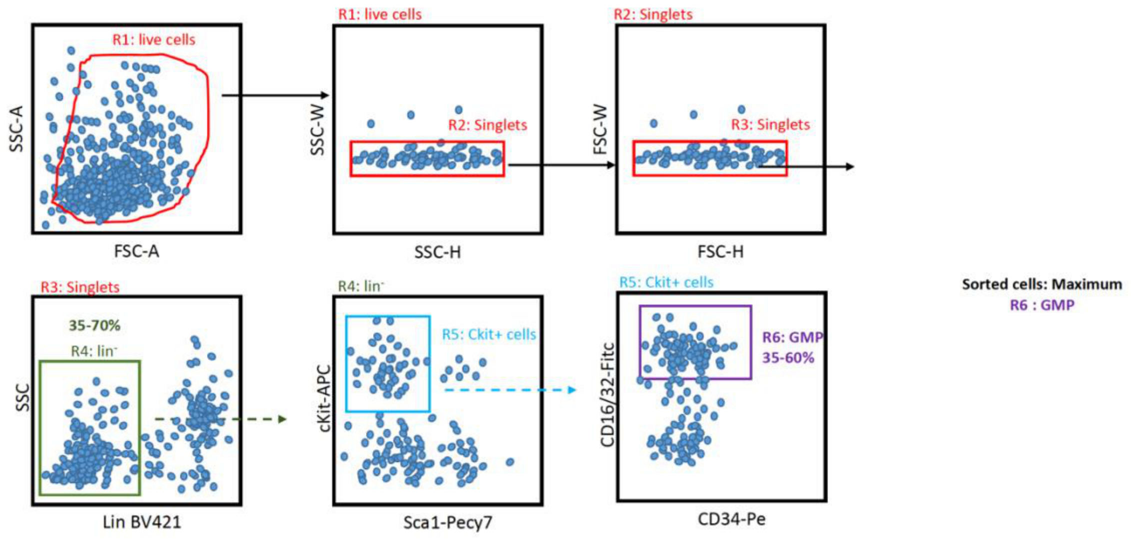

To obtain GMPs : FSC-A/SSC-A gating of bone marrow cells was done to obtain cells in gate R1; further gating through SSC-W/SSC-H and FSC-W/FSC-H to obtain singlets in gate R3 removing any non-single cells such as doublets, clumps or debris. Mouse lineage cell depletion kit (Miltenyi Biotec) was used to establish gate R4 (against SSC). Cells from R4 were gated by staining for c-KIT and SCA1 and c-KIT+, SCA-1- cells were gated by staining with antibodies against CD16/32 versus CD34 to obtain gate R6, which represent GMPs.

Suppl. Fig. 12

Gating strategy for isolating preNeu and matNeu cells from bone marrow

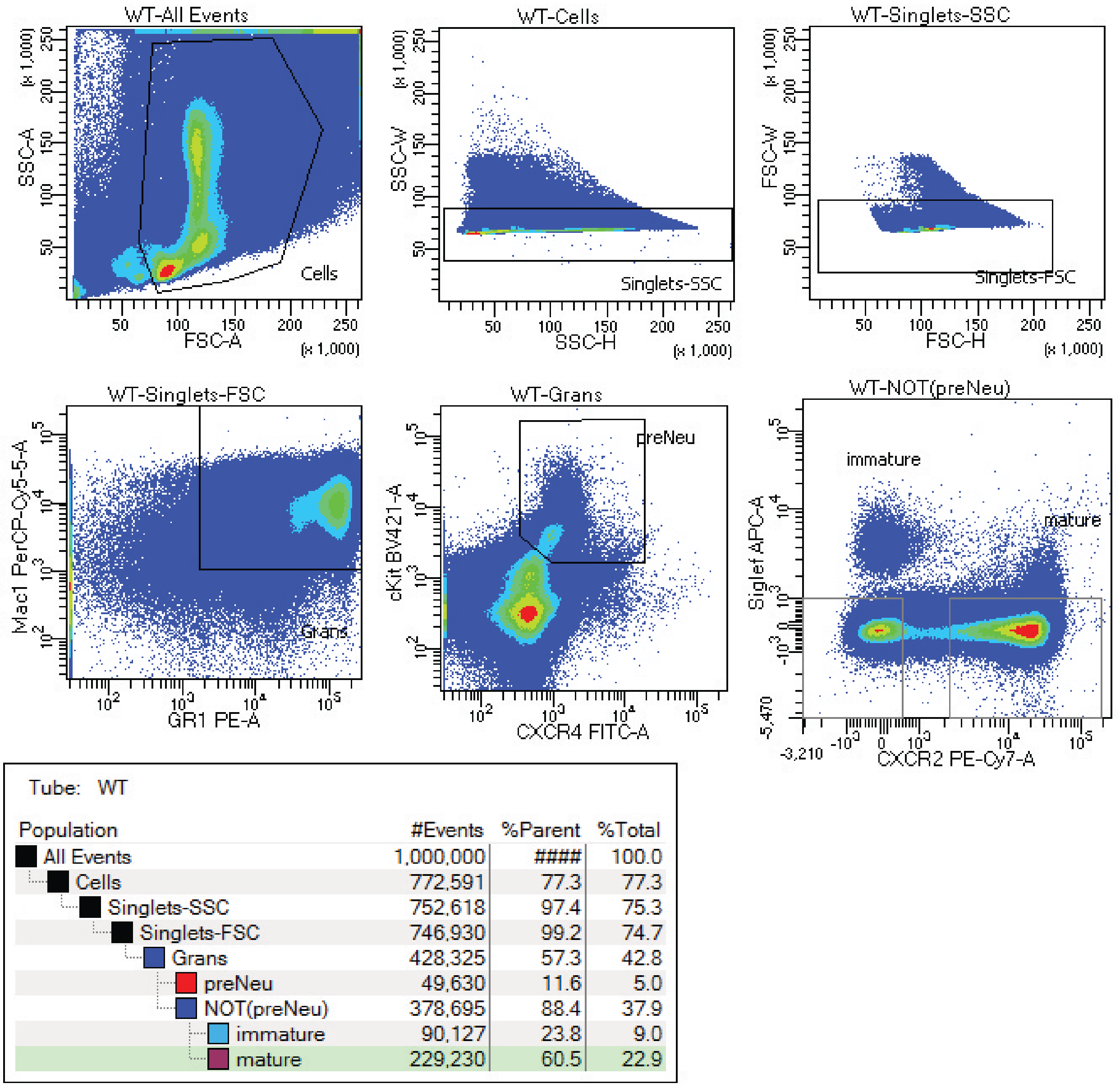

Tube: WT

Population	#Events	%Parent	%Total
All Events	1,000,000	####	100.0
Cells	772,591	77.3	77.3
Singlets-SSC	752,618	97.4	75.3
Singlets-FSC	746,930	99.2	74.7
Grans	428,325	57.3	42.8
preNeu	49,630	11.6	5.0
NOT(preNeu)	378,695	88.4	37.9
immature	90,127	23.8	9.0
mature	229,230	60.5	22.9

preNeu: **4 000 000 x 1 tube** **+ 1 097 989 cells**
immature: **4 000 000 X 3 tubes** **+ 505 870 cells**
mature: **4 000 000 x 6 tubes** **+ 1 031 714 cells**

To obtain pre neutrophils (PreNeu) and mature neutrophils (MatNeu): As for GMP sorting, FSC-A/SSC-A gating of bone marrow cells was done to obtain cells in gate R1; further gating through SSC-W/SSC-H and FSC-W/FSC-H to obtain singlets in gate R3 removing any non-single cells such as doublets, clumps or debris. Next, Mac-1+, Gr-1 + were gated on R3 to obtain granulocytes. This population was further gated to obtain cKIThi, CXCR4med cells and then the mature neutrophil population that is Siglecf-, CXCR2+.

Suppl. Fig. 13

Whole Western blots for suppl. Fig. 5b

GFI1

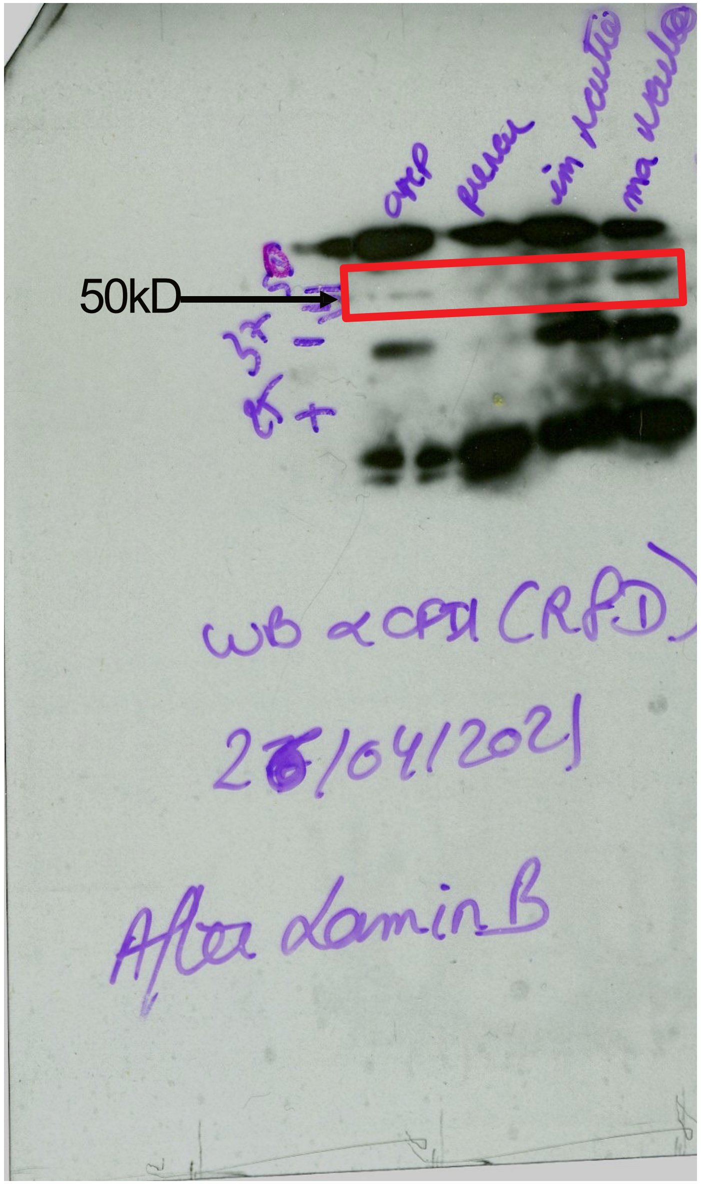

Lamin B

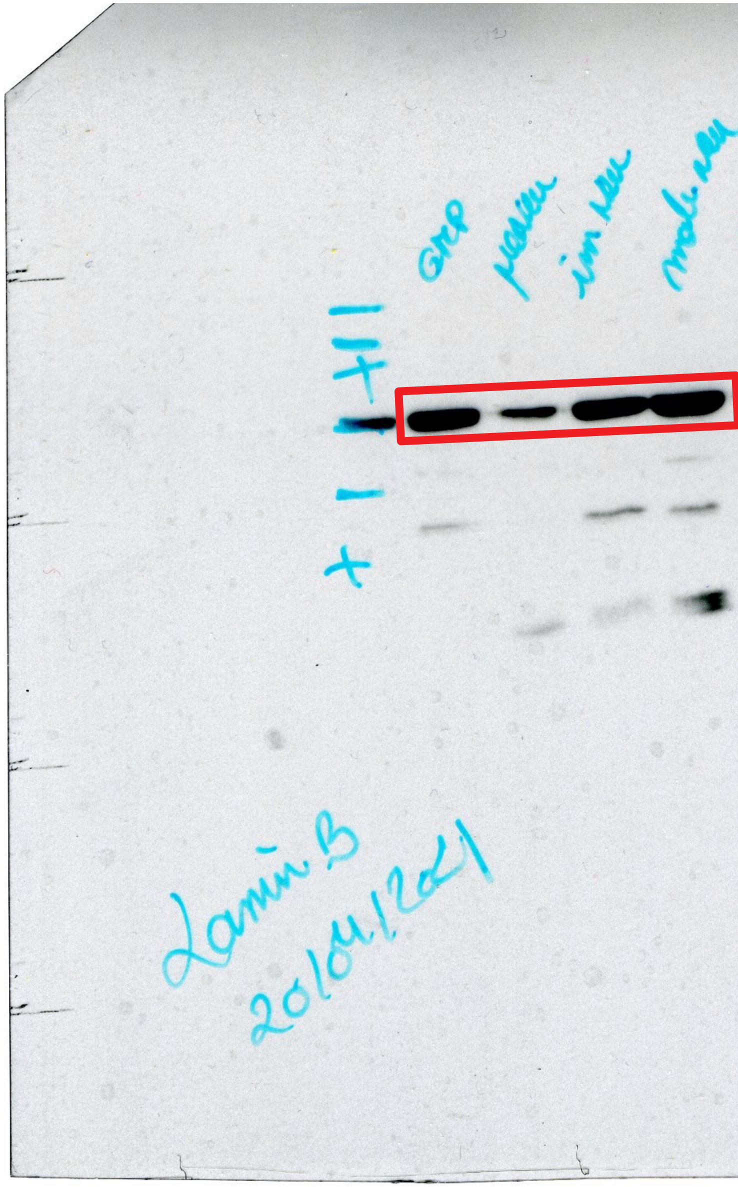

Suppl. Table 1

group1	group2	pvalue	fdr	
H3K4me2_GMP_CHD4	H3K4me2_GMP_CHD4/Gfi1	0,388959964	0,400073106	**Promoter**
H3K4me2_GMP_CHD4	H3K4me2_GMP_Gfi1	3,0832E-05	9,2496E-05	
H3K4me2_GMP_CHD4/Gfi1	H3K4me2_GMP_Gfi1	0,00268003	0,004824053	
H3K4me2_PreNeu_CHD4	H3K4me2_PreNeu_CHD4/Gfi1	0,024944722	0,029933666	
H3K4me2_PreNeu_CHD4	H3K4me2_PreNeu_Gfi1	1,98228E-09	1,01946E-08	
H3K4me2_PreNeu_CHD4/Gfi1	H3K4me2_PreNeu_Gfi1	2,88873E-07	1,15549E-06	
H3K4me2_MatNeu_CHD4	H3K4me2_MatNeu_CHD4/Gfi1	0,00754659	0,012348966	
H3K4me2_MatNeu_CHD4	H3K4me2_MatNeu_Gfi1	0,019114389	0,026466077	
H3K4me2_MatNeu_CHD4/Gfi1	H3K4me2_MatNeu_Gfi1	0,000110681	0,000267533	
ATACSeq_GMP_CHD4	ATACSeq_GMP_CHD4/Gfi1	5,45645E-05	0,000151102	
ATACSeq_GMP_CHD4	ATACSeq_GMP_Gfi1	1,16249E-32	1,39499E-31	
ATACSeq_GMP_CHD4/Gfi1	ATACSeq_GMP_Gfi1	0,021706006	0,028382655	
ATACSeq_PreNeu_CHD4	ATACSeq_PreNeu_CHD4/Gfi1	0,000111472	0,000267533	
ATACSeq_PreNeu_CHD4	ATACSeq_PreNeu_Gfi1	1,83812E-43	3,30862E-42	
ATACSeq_PreNeu_CHD4/Gfi1	ATACSeq_PreNeu_Gfi1	0,00030706	0,000650244	
ATACSeq_MatNeu_CHD4	ATACSeq_MatNeu_CHD4/Gfi1	0,000971985	0,00194397	
ATACSeq_MatNeu_CHD4	ATACSeq_MatNeu_Gfi1	1,01358E-45	3,64888E-44	
ATACSeq_MatNeu_CHD4/Gfi1	ATACSeq_MatNeu_Gfi1	2,95617E-06	9,67472E-06	
				**Enhancer**
H3K4me2_GMP_CHD4	H3K4me2_GMP_CHD4/Gfi1	0,022075398	0,028382655	
H3K4me2_GMP_CHD4	H3K4me2_GMP_Gfi1	1,1483E-17	6,88981E-17	
H3K4me2_GMP_CHD4/Gfi1	H3K4me2_GMP_Gfi1	0,000274254	0,000617071	
H3K4me2_PreNeu_CHD4	H3K4me2_PreNeu_CHD4/Gfi1	0,001469442	0,002784206	
H3K4me2_PreNeu_CHD4	H3K4me2_PreNeu_Gfi1	5,39382E-28	3,88355E-27	
H3K4me2_PreNeu_CHD4/Gfi1	H3K4me2_PreNeu_Gfi1	1,60435E-06	5,77566E-06	
H3K4me2_MatNeu_CHD4	H3K4me2_MatNeu_CHD4/Gfi1	0,005913535	0,010137489	
H3K4me2_MatNeu_CHD4	H3K4me2_MatNeu_Gfi1	2,18322E-28	1,9649E-27	
H3K4me2_MatNeu_CHD4/Gfi1	H3K4me2_MatNeu_Gfi1	1,10396E-08	4,96783E-08	
ATACSeq_GMP_CHD4	ATACSeq_GMP_CHD4/Gfi1	0,012029081	0,018828127	
ATACSeq_GMP_CHD4	ATACSeq_GMP_Gfi1	0,408054971	0,408054971	
ATACSeq_GMP_CHD4/Gfi1	ATACSeq_GMP_Gfi1	0,091715442	0,103179872	
ATACSeq_PreNeu_CHD4	ATACSeq_PreNeu_CHD4/Gfi1	0,013266204	0,019513086	
ATACSeq_PreNeu_CHD4	ATACSeq_PreNeu_Gfi1	0,024441512	0,029933666	
ATACSeq_PreNeu_CHD4/Gfi1	ATACSeq_PreNeu_Gfi1	0,369143642	0,390857973	
ATACSeq_MatNeu_CHD4	ATACSeq_MatNeu_CHD4/Gfi1	0,013550754	0,019513086	
ATACSeq_MatNeu_CHD4	ATACSeq_MatNeu_Gfi1	0,077456047	0,089948958	
ATACSeq_MatNeu_CHD4/Gfi1	ATACSeq_MatNeu_Gfi1	0,313178777	0,341649574	

Supplementary Table 1
P-values and FDR values for Metagene analysis

Suppl. Table 2: Compilation of functional enrichment analyses using gprofiler https://biit.cs.ut.ee/gprofiler/gost.

CHD4 occupied genes:	GFI1 occupied genes:	CHD4 and GFI1 occupied genes:	CHD4 and GFI1 occupied genes:
**downregulated, Immune system process**	**downregulated, Metabolic processes**	**downregulated, Nucleosome assembly**	**downregulated, Cellular metabolic process**
RIM38, VAV3, CTSH, PRKCQ, INPP4B, TNFAIP8, ITGA4, REST, RAP1A, PIK3R1, MRC1, GBP5,	ERC1, LARP4B, MORC3, MAP2K2, ATXN1, ANKRD13A, RSF1, VTI1A, HERC3, PIP5K1A, APAF1, NIPBL, APLP2, RIPK2, GIT2, ZFP263	Hist1h4h, Hist1h3e, Hist1h2bp, Hist1h3a, Hist1h3i, Hist1h2bm, Hist1h4d, Hist1h4j, Hist2h3c2, Hist1h4k, Hist1h3g, Hist1h3d, Hist1h1b	VAV3,HAL,PHIP,TNFRSF14,TAX1BP1,RAP1A,DHRS3,DGLUCY,RBPMS,DHX8,CLTC,ENC1,DSTYK,EIF4A2,SGK3,ETV6,RNASE4,GTF2A1
**upregulated, Immune system process**	**upregulated, Metabolic processes**	**upregulated, Nucleosome assembly**	**upregulated, Immune system process**
SIRPB1C, SIRPB1A, FPR2, SIRPB1B, CD300LD, ITGAX, LILRB4A, CXCL2, PLA2G7, BST1, S100A8, CLEC4D, WFDC17, TLR13	PROK2, IER5, DUSP1, GADD45G, TRIB1, BTG1, PAPLN, ID3, APOBR, JUNB, BTG2, ARSG, KLF6, JUND, PBXIP1, TRP53INP2, CEBPD, ARID3B	Hist1h2br, Hist1h2bb, Hist1h3c, Hist1h1c, Hist1h4n, Hist1h3h, Hist1h4c, Hist1h2be, Hist1h1e, Hist2h3b, Hist1h3f, Hist1h2bj, Hist1h4i, Hist4h4	TRIM38,VAV3,CTSH,PRKCQ,INPP4B,TNFAIP8,ITGA4,REST,RAP1A,PIK3R1,MRC1,GBP5,IL15RA,RAB29,GPR18,NLRC5,FZD

Supplementary Table 2

Genes occupied by CHD4, GFI1 or both CHD4 and GFI1 and were up or downregulated from GMP to matNeu cells were mapped the Gene Ontology, biological function database. Shown are the top 6 or 7 GO groups according to their adjusted p value. Below the representation of the GO pathways, a short list of examples of genes is shown.

Suppl. Table 3
List of oligonucleotides

Primers for CHD4CHIP-qPCRon WT and gfi1 KO GMPs

Gene	Forward	Reverse
CD34 promoter	GGAGAGCCAATATCCCCCAC	CTGCATTCTCCTGTACCGCT
CD34 intron	AGCGACTTGTGGGACTTTGT	GCCCTTCCTTCTGCTTTTGC
Csf1r	GTGAGAGCCCAAGTGTCGAA	GTGTGGGCGGAAACACATTG
Mmp8	GCTTTGTTGAATGACTCCCCC	GAGGCAACATAGCTAACCGCA
Cfp	AGGGTGACAAAGCAGCTACA	TGGGAGGGTATGGCCTCATT
Hal	TTGCTCAACGCGTGTTTGT	TGGTAAAACGCATCCTCTCGG
Jun	AAGAAGCTCACAAGTCCGGG	TTGTTACCGGTCCTCTGGGT
Rhobtb1	CTCGTCTCGTTGCAGGACTA	CTAACTTGGCTCCTGTCCGC
Fcgr4 enhancer	TCAAATGTACGCTAAGAAGTTGGT	TCAACCTACATTTTCTTTCAAAACG
Fcgr4 promoter	AGGAAACTGGTGAGATGGACT	ACCACAGATATCCGGAACCC
HOxa9	TCATAATTTCGGCGGGTCGG	CAGCAGGAAGGAGTCCACAT
Meis 1	CCACCACTACTTCCGGGTTC	CCGTGCGTGTGTAAAGTGTG

Primers for Expression analysis WT and Gfi1 KO GMPs

Gene	Forward	Reverse
Cd34	TGATGAACCGTCGCAGTTGG	CTTCCCAACAGCCATCAAGG
Csf1r	CTTGGGAGCCTGTACTCACG	CTAGCATAGCCTCGGCCTTC
Csf1	CCTTCTTCGACATGGCTGGG	GTTCTGACACCTCCTTGGCA
Ebf1	TGCCATCCGAGTTCAGACAC	ATCTGCCTGGTGTCCCTTTG
Gapdh	ACTGAGCAAGAGAGGCCCTA	TATGGGGGTCTGGGATGGAA

Primers for the detection of Mycoplasma contamination

	Forward	Reverse	
Myco-5-1	CGCCTGAGTAGTACGTTCGC	GCGGTGTGTACAAGACCCGA	Myco-3-1
Myco-5-2	CGCCTGAGTAGTACGTACGC	GCGGTGTGTACAAAACCCGA	Myco-3-2
Myco-5-3	TGCCTGAGTAGTACATTCGC	GCGGTGTGTACAAACCCCGA	Myco-3-3
Myco-5-4	TGCCTGGGTAGTACATTCGC		
Myco-5-5	CGCCTGGGTAGTACATTCGC		
Myco-5-6	CGCCTGAGTAGTATGCTCGC		

Suppl. Table 4
List of antibodies

	Name	code	company
GranulocytesortingStreptavidin	nanobeads	480016	Biolegend
	Gr1-PE	553128	BD
	Mac1-PerCP/Cy5.5	101228	Biolegend
	ckit-bv421	105828	Biolegend
	cxcr4-fitc	551967	bd
	Siglec-F-APC	155508	Biolegend
	Cxcr2-PE/Cy7	149316	Biolegend
GMP	CD16/32	553144	BD
	Streptavidin PE-Cy5	15-4317-82	eBioscience
	ckit-APC	105812	Biolegend
	CD34-PE	119308	Biolegend
	Sca1-PECy7	558162	BD
Lineage depletion	CD3	51-01082J	BD
	CD8	100704	Biolegend
	CD4	100508	Biolegend
	B220	103204	Biolegend
	IgM	553406	BD
	Gr1	51-01212J	BD
	Mac1	51-01712J	BD
	Ter119	51-09082J	BD
	NK1.1	13-5941-85	Invitrogen
	CD5	13-0051-85	eBioscience
	DX5	553856	BD
	CD115	13-1152-82	eBioscience
	Sca1	553334	BD
intern. staining CHD4	CHD4-PE	NBP2-50163PE,	Novus
	MBD3 (C-18)	sc-9402	Santa cruz
	MBD2/3	sc-271562	Santa cruz
	Rbap46/48	4633S	Cell signaling
	Rbap46/48	ab79416	abcam
	MTA3	IM1012	Calbiochem
	MTA2	ab8106	abcam
	MTA2	ab50209	abcam
	CHD4	ab70469	abcam

Suppl. Table 4 (continued)

List of antibodies

	CHD4/Mi2	sc-11378	Sanat Cruz (Discontinued)
	HDAC1	ab7028	abcam
	HDAC2		
	LSD1	ab17721	abcam
	CoREST	sc-30189	Sanat Cruz
Co-IP beads	anti-flag M2	F1804	Sigma
	IgG	PP64B	Millipore
	IgG	ab46540	abcam
	IgG	ab18413	abcam
	H3 total	ab1791	abcam
	H3K4me2	ab7766	abcam
	H3K4me2	710796	invitrogen
	H3K4me3	ab8580	abcam
	H3K27ac	ab4729	abcam
	H3K27me3	07-449	millipore
	H3K9me3	ab8898	abcam
Fixing agent	FA	F8775-25ml	Sigma
	DSG	20593	Thermo
	Gfi1	singh lab	in house
	Gfi1	ab21061	abcam
	Gfi1	AF3540	R&D
	Gfi1 (B-9)	sc-376949	SAnta Cruz
	Gfi1 (N-20)	sc-8558	SAnta Cruz
	Gfi1 (15-8)	sc-101053	SAnta Cruz
	Gfi1(H-200)	sc-22796	SAnta Cruz
Western kit	34076	Thermo fisher	
	anti-mouse HRP	115-035-174Jackson	ImmunoResearch
	anti rabbit HRP	211-032-171Jackson	ImmunoResearch
	anti-goat HRP	205-032-176Jackson	ImmunoResearch

Suppl. Table 5

Samples used in experiments for Figs. 6, 7, and suppl. Fig.s 5–7

In RLT buffer for PCR

Pre neutro KO 1	Around 500 000 cells
Pre neutro KO 2	Around 500 000 cells
Immature neutro KO 1	Around 500 000 cells
Immature neutro KO 2	Around 500 000 cells
Immature neutro KO 3	Around 500 000 cells
Pre neutro WT 1	Around 500 000 cells
Pre neutro WT 2	Around 500 000 cells
Immature neutro WT 1	Around 500 000 cells
Immature neutro WT 2	Around 500 000 cells
Immature neutro WT 3	Around 500 000 cells
Mature neutro WT 1	Around 500 000 cells
Mature neutro WT 2	Around 500 000 cells
Mature neutro WT 3	Around 500 000 cells

Cytoplasmic and Nuclear protein extracts

Pre neutro WT 1	around 8 millions of cells
Immature neutro WT 1	around 12 millions of cells
Mature neutro WT 1	around 25 millions of cells
Immature Neutro KO	around 8 millions
