# Supplementary material for: GFI1 tethers the NuRD complex to open and transcriptionally active chromatin in myeloid progenitors
Source: Commun Biol. 2021 Dec 2;4:1356. doi: 10.1038/s42003-021-02889-2 (PMC8639993; doi:10.1038/s42003-021-02889-2)
Supplement: Supplementary file 3 — Description of Additional Supplementary Files [file 42003_2021_2889_MOESM3_ESM.pdf]

# Description of Additional Supplementary Files

**File name:** Supplementary Data 1.
**Description:** Unfiltered SAINT result of the GFI1_WT, GFI1_DeltaSNAG and GFI1B BioID data.

**File name:** Supplementary Data 2.
**Description:** GO term analyses including biological processes using KEGG and CORUM databases.
